# The development and validation of the assessment engagement scale

**DOI:** 10.3389/fpsyg.2023.1136878

**Published:** 2023-06-27

**Authors:** Carol Evans, Xiaotong Zhu

**Affiliations:** ^1^Learning and Teaching Academy, Cardiff University, Cardiff, United Kingdom; ^2^Eleanor Glanville Institute, University of Lincoln, Lincoln, United Kingdom

**Keywords:** student engagement, assessment, instrument validation, self-regulation, higher education, student success

## Abstract

**Introduction:**

The quality of student engagement in assessment within higher education affects learning outcomes. However, variations in conceptions of what quality in engagement looks like impacts assessment design and the way that students and lecturers engage with each other in the assessment process. Given that assessment is an important driver of student engagement in higher education, it is surprising that no specific measures to support understanding of this measure exist. To address this significant gap, we outline the evolution of an assessment engagement scale derived from a research-informed conceptual framework utilizing best practice in assessment and feedback.

**Methods:**

We consider the validity and utility of the assessment engagement scale in supporting students’ understanding of assessment and their role within it using exploratory and confirmatory factor analyses.

**Results:**

The resultant nine-item assessment engagement scale’s underpinning two factors included: (i) *Understanding of the Assessment Context* (UAC) including one’s role within it, and confidence in navigating assessment requirement, and (ii) *Realising Engagement Opportunities* (REO) (i.e., willingness to engage and ability to utilise the assessment context effectively to support one’s understanding). Construct, criterion, and convergent validity of the scale were established.

**Discussion:**

The AES is a powerful tool in promoting dialogue between lecturers and students about what high quality engagement in assessment looks like, and the respective roles of all parties in realising this. Implications for assessment practices are discussed along with the potential of the scale as a predictive and developmental tool to support enhancements in assessment design and student learning outcomes in higher education.

## 1. Introduction

This article discusses the development and validation of the Assessment Engagement Scale, within higher education. Creating learning environments that promote meaningful student engagement is essential to support student learning (Marôco et al., 2016; Chipchase et al., 2017; Boulton et al., 2019). Meaningful engagement in assessment impacts students’ attainment and development of self-regulatory skills (Ibarra-Saiz et al., 2020; Musso, 2020). However, the relationship between student success and engagement is complex given the multi-dimensional nature of the construct, and the many variables implicated (Dalrymple et al., 2014; Esposito et al., 2021; Trowler et al., 2021). Engagement is impacted by differing conceptions of what the student role in assessment is by both students and lecturers alike.

While engagement has become a “catch-all term” to describe how students engage with their university contexts, there is a need for discernment in how engagement is defined and encouraged (Krause, 2005). Engagement needs to focus on those activities that promote students’ self-regulatory skills (Luo and Gan, 2022) both within the immediate requirements of an assessment context and beyond it (Evans, 2022).

Clarity is needed on what discerning approaches to student engagement look like in 21^st^ century learning environments within specific disciplines if we are to cultivate these *with* our students. We need to be explicit about the scope, intent, and parameters of engagement, and robust in our measurement of it if we are to maximize the potential of engagement to support student learning.

Assessing student engagement in higher education is challenging given the lack of understanding, and explicit instruction in how best to promote it (Mandernach, 2015). Assessment is a key driver of student engagement in learning (Dawson et al., 2019), however, there are no measurement tools that specifically focus on supporting student engagement in assessment within higher education which makes the focus of this article especially pertinent.

There are numerous survey tools including the National Survey of Student Engagement (NSSE, 2016) used in the US and Canada, the UK Engagement Survey (UKES, 2013), and the Australasian Survey of Student Engagement (AUSSE) (Mandernach, 2015; Chipchase et al., 2017) that support generic understandings of student engagement. However, these surveys tell us little about the process of learning, the ways in which students engage in assessment practices and processes, and provide little support to students around what meaningful engagement looks like.

Given the importance of the design of assessment on how students engage in learning (Evans, 2013), there is a need for clearer understanding of student engagement (Evans et al., 2015). Student engagement as an outcome measure is frequently noted in reviews of assessment (Pitt and Quinlan, 2021), but the term is insufficiently critiqued. Discernment is needed in relation to normative assumptions that any form of pedagogical activity that gets students involved in learning is a good thing. Scrutiny is needed around the form and quality of activities that promote engagement and especially when considering students with diverse needs. Being actively involved is not the same as being engaged, and so-called engaged students may still undertake “surface learning” (Marton and Saljo, 1976), utilize their time and skillsets inappropriately (Schneider and Preckel, 2017) and/or experience learner “alienation” (Mann, 2001). A key consideration is what constitutes purposeful engagement in assessment that impacts students’ learning (Coates, 2007; Kuh, 2008), and what tools can best support this understanding. With this in mind, we report on the development and validation of the Assessment Engagement Scale to support meaningful student engagement in assessment practice in higher education.

## 2. Student engagement: a multi-dimensional construct

Qualitatively different conceptions of student engagement exist ranging from single dimensional interpretations focused on behaviors (e.g., time on task) to multi-dimensional constructs such as “student ownership of learning” (Evans et al., 2015). Multi-dimensional models of engagement including learner dispositions (e.g., motivations, emotions, self-regulation) are important given that these variables dynamically influence engagement and academic performance over time (Tempelaar et al., 2018; Boulton et al., 2019). Such models commonly comprise a range of cognitive, affective, and metacognitive dimensions of engagement (Chapman, 2002), albeit with different foci. Marôco et al.’s (2016) *University Student Engagement Inventory* (USEI) focuses on behavioral, psychological and holistic aspects of engagement, while Collie and Martin (2021) integrate motivation and engagement constructs. Greater emphasis is now placed on the nature of student engagement in the learning process (Trowler, 2016), and the importance of dialogic processes to support student understanding of assessment and their role in it within higher education (Sadler, 1989, 2021; Boud, 2000; Carless, 2011; Evans, 2013, 2016; Nicol, 2022).

Increasing discernment is also evident around discussions of student engagement. For example, (i) the limitations of using behavioral data (i.e., the number of times a student accesses a virtual learning environment) as an indication of quality engagement (Holmes, 2018; Boulton et al., 2019); (ii) enhanced acknowledgement of cognitive and emotional dimensions of engagement (Luo and Gan, 2022). Of profound importance is what environmental and student engagement variables matter most, and in what particular contexts and for whom (Evans and Waring, 2021).

While recent definitions of student engagement incorporate the role of higher education institutions (Holmes, 2018), much of this work lacks specificity. The reciprocal responsibility of both students and institutions in fostering engagement is highlighted (Kuh, 2003; Evans, 2013). Evans and Waring (2021) identified the central role of assessment design in impacting how students engage cognitively, affectively, and metacognitively in assessment while also mindful of the role of individual differences in framing responses.

Temporal, spatial, and discipline-specific elements impact student engagement given the fluidity of the construct and the context-and individual differences-dependence nature of it. Students may choose to engage in certain activities and not others (Trowler, 2016; Chipchase et al., 2017). Lack of engagement may reflect student disengagement and alienation (Mann, 2001), but could also reflect students’ high level self-regulatory skills in attending to areas that matter most to them (Schneider and Preckel, 2017).

In seeking to delve deeper into what student engagement for the 21st century looks like, most would agree that engagement is dynamic involving an interactional space between a learner and all resources within and beyond an immediate learning context (Trowler et al., 2021). Engagement comprises dynamic and static elements in that while engagement is located in the moment, it is also informed by previous experiences of learning that can trigger powerful responses (Price et al., 2011). Agentic engagement, the capacity of an individual to influence their learning context and make it work better for them (Reeve, 2013) is key to meaningful engagement. Agentic engagement is about student-initiated, proactive, intentional, collaborative, and constructive action to support their learning and how as educators we best support this (Evans and Waring, 2021, p. 30).

Students’ self-regulatory skills are crucial to impactful engagement. This includes being able to manage the cognitive and emotional demands of a task, dependent on students’ metacognitive capacity in knowing what to focus on, what strategies to deploy and when, in order to achieve goals (Chapman, 2002; Evans et al., 2019). In seeking understanding of what constitutes quality of engagement, the use of deep approaches to learning (McCune and Entwistle, 2011), choice of the right strategies and good execution of them is crucial (Dinsmore, 2017; Van Merrienboer and de Bruin, 2019). Higher levels of student engagement require greater levels of intrinsic motivation, cognitive and metacognitive thinking about what one is doing, purposeful pursuit of goals, and owning and valuing learning (Harris, 2008).

While it is accepted that there are disciplinary and temporal variations in what a deep approach to learning looks like, there is broad consensus that it includes: (i) the intention to understand and construct the meaning of the content to be learned (Asikainen and Gijbels, 2017), (ii) the ability to apply and adapt such understandings in the creation of useful outputs which includes the notion of transfer (Evans et al., 2015), (iii) sensitivity to the requirements of a context which includes noticing skills (Van Merrienboer and de Bruin, 2019), and (iv) a willingness to contribute by offering up ideas for scrutiny by others (McCune and Entwistle, 2011).

A modern take on a deep approach within the assessment context of higher education is synonymous with intelligent use of resource: students’ deployment of metacognitive skills in knowing where and how to invest efforts to best effect (Dinsmore, 2017; Schneider and Preckel, 2017). Participation alone does not in itself imply engagement (Chipchase et al., 2017). Effective and efficient engagement includes students’ accurate appraisal of task requirements, setting of appropriate goals, choice and deployment of strategies, accurate reading of the context and how to utilize the environment effectively to support learning, effective monitoring of progress, and self-evaluative capacity of the extent to which work meets the standards required; it relies on good use of self-regulatory skills (Zimmerman, 2000; Eva and Regehr, 2011).

Engagement is dependent on students’ assessment literacy (the skills, knowledge and dispositions implicated in coming to know what the requirements of assessment are) (Pastore and Andrade, 2019). From an individual differences perspective, students’ understanding of their assessment context is impacted by their cognitive processing styles (Kozhevnikov et al., 2014), previous experiences of learning, and a whole suite of individual difference variables that fall within the self-regulation umbrella (Panadero, 2017). Student engagement in assessment, therefore, requires an integrated approach, given that cognitive factors impact how individuals process information (Friedlander et al., 2011), socio-cultural factors affect how individuals engage with others, and socio-political factors impact how individuals navigate the many cultures of assessment (James, 2014).

How assessment is designed to enable meaningful engagement from lecturers and students is critical (Evans, 2013). For example, student disengagement from certain assessment activities (e.g., inaccessible content, insufficient challenge, feedback too late to be of value, feedback that does not feedforward, feedback that may cause harm) may reflect utilization of high level self-regulatory skills, in students’ discerning accurately, that for them, such activities have little value in enhancing their learning (Chipchase et al., 2017; Vattøy et al., 2021). Overemphasis on certain approaches to student engagement may be counterproductive. For example, Sadler (1989, 2009) has questioned the obsession with “*why students do not engage with feedback*”, arguing that while student understanding of feedback and use of it is important, receipt of feedback from others is not the best way to promote students’ internalization of what quality looks like (Sadler, 2021). Instead, focus needs to be on promoting those self-regulatory practices that enable students to develop their internal feedback capabilities (Evans et al., 2019; Nicol, 2022).

Evans (2016, 2022) in her integrated assessment framework (EAT), brings together understanding of individual differences, agentic engagement, and self-regulation, to consider how individuals shape and are shaped by the contexts in which they are operating (Pintrich, 2004). Emphasis is placed on how individuals perceive and process information, and develop the skill-sets to manage assessment for themselves, marking a significantly different emphasis to generic models of student engagement (Kahu, 2013). The EAT Framework aligns with Kahu et al.’s (2019) holistic conception of engagement as encompassing behavioral, psychological, and socio-cultural approaches but differs in its interactional emphasis and focus on assessment. Engagement in Evans’ assessment framework is seen as interactionist, reciprocal and dynamic (James, 2014; Trowler et al., 2021).

In sum, meaningful student engagement in assessment is a multi-dimensional construct that is highly situated in terms of the nature of learner interaction within a specific learning context. Such engagement requires self-ownership of a learning situation. It involves the confidence and ability to manage one’s learning environment and adapt it to address one’s learning needs (Reeve, 2013). This is achieved through the effective and *combined* use of self-regulatory skills to include metacognitive (strategic), cognitive (processing), and affective skills (management of emotions) that enable learners to select the most appropriate strategies (Vermunt and Donche, 2017), and to deploy them accurately in meeting the requirements of a task/situation (Dinsmore, 2017). State and trait-like qualities of engagement are included in this definition in that some individuals may have more inherent flexibility than others in being able to adapt their learning to the requirements of different contexts (Kozhevnikov et al., 2014).

To better understand and support the development of student engagement in assessment, in the next section we outline the processes involved in constructing and validating the Assessment Engagement Scale. We explore the validity and reliability of the instrument to include face, construct, predictive, and convergent validity, and implications for practice and research based on extensive use of the tool across varied contexts (discipline, institution).

## 3. Theoretical framing

The Assessment Engagement Scale was derived from the Equity, Agency and Transparency Assessment (EAT) Framework (Evans, 2016, 2022) given its emphasis on promoting self-regulatory skills development of lecturers and students within assessment. The theoretical basis of EAT combines constructivist, socio-cultural, and socio-critical theoretical perspectives to facilitate understanding of student and lecturer engagement in the assessment process. The Framework was derived from systematic analysis of over 50,000 articles.

From a constructivist perspective the Framework draws attention to the internal processes students use to manage their learning including consideration of their conceptions of, and approaches to learning, and preferred ways of processing information (Waring and Evans, 2015). Socio-culturally, EAT explores how individuals shape and are shaped by the learning environment (Bandura, 1986). Agentic engagement, learner intentionality, willingness, and ability to influence the learning context (Reeve, 2013) links with self-regulation (Dinsmore, 2017) and self-determination theories concerned with competence, autonomy, and relatedness (Ryan and Deci, 2000). Drawing on a critical pedagogy perspective EAT asks who is privileged and disadvantaged by the way in which assessment is designed (Butin, 2005).

**Figure 1:**
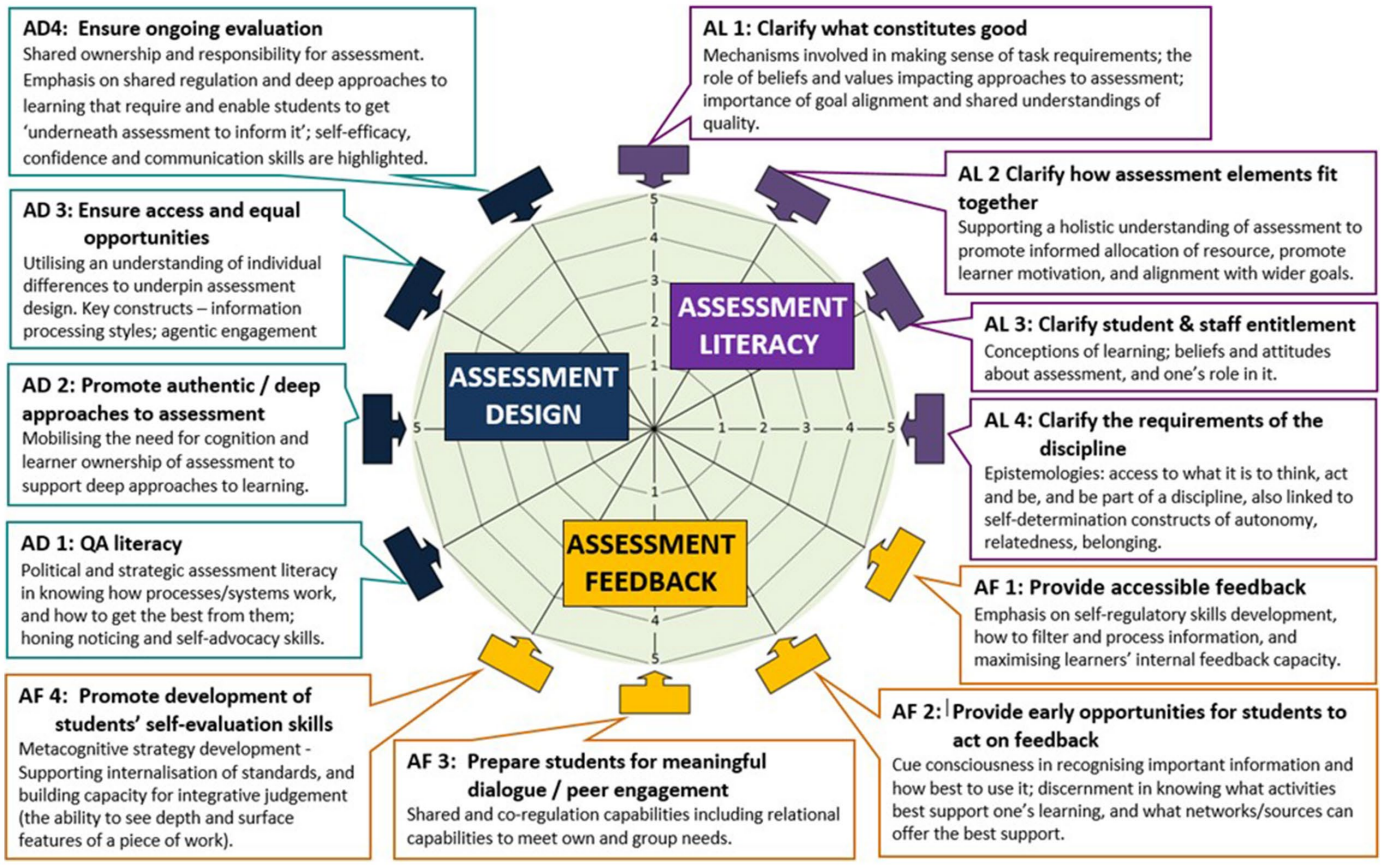
Core themes underpinning the student version of the assessment engagement scale.

Conceptually, the EAT Framework identifies three key interrelated assessment dimensions: assessment literacy, feedback and design, with twelve sub-dimensions in total making up the framework as depicted in Figure 1. Core concepts integral to the EAT Framework include inclusivity, and partnership between academics and students to support agency and autonomy in assessment practices. As demonstrated in the Supplementary Table S1 each sub-dimension of the framework brings together understandings of autonomy supportive assessment design that is informed by an understanding of individual differences in supporting students’ development and use of self-regulatory skills.

## 4. Materials and methods

### 4.1. Aims

Our overarching aim was to develop an Assessment Engagement Scale *(AES)* with sound psychometric properties, and ease of use for academics and students within higher education; mindful of this intention we focused on the following four objectives:

- Objective 1 (01): To clarify the *AES*’s factor structure by confirming the factors underpinning the scale.
- Objective 2 (02): To establish the criterion validity of the *AES.*
- Objective 3 (03): To establish the convergent validity of the *AES.*
- Objective 4 (04): To confirm the utility of the *AES.*

### 4.2. Research context

The Assessment Engagement Scale was developed and tested within the context of a UK Russell group university (23,000 students) that had embarked on a significant program of developing and implementing research-informed assessment practices. Research funding facilitated a comprehensive series of longitudinal pedagogical assessment interventions to support student and lecturer engagement in assessment. Pre-and post-intervention data collected as part of this research was used to test the validity of the Assessment Engagement Scale, one of the instruments used to support understanding of student and lecturer engagement in assessment (Evans et al., 2019). Academic and professional services staff and students from across all faculties of the University participated in, and contributed to, extensive training opportunities (e.g., >190 training events over 3–4 years) to promote shared understandings of effective assessment practices and the relevant theoretical positions informing them. In this article we focus on the student version of the scale but identify that both lecturer and student versions were used extensively to promote a research-informed approach to assessment engagement. Starting with the lecturer version of the Assessment Engagement Scale was an essential first step in supporting lecturer confidence and competence in supporting students’ self-regulatory approach to assessment.

The Assessment Engagement Scale was used in many ways to support both student and lecturer engagement in assessment: (i) as a *diagnostic tool* to explore gaps and priorities, and areas of strength and weaknesses in assessment; (ii) as a *design tool* to support shared understandings of principles of effective assessment and feedback; (iii) as an *evaluative tool* to build quality at all levels within the organization; (iv) as a *predictive tool* to identify variables impacting student success and the relationships between them such as the relationships between students’ perceptions of engagement and outcomes (satisfaction, use of effective strategies, grades etc.).

### 4.3. Participants

The sample for this study comprised two groups of undergraduate students from one UK Russell group university (Table 1). The sampling frame was stratified in engaging with different faculties, purposeful in engaging with key assessment leads within the university, and opportunistic in working with students who had elected to be involved in the initiative.

**Table 1:** Participant profile.

	Pre-intervention participant (sample one)			Post-intervention participant (sample two*)		
Demographic characteristics	Frequency (%)	Mean (SD)	Range	Frequency (%)	Mean (SD)	Range
Total	453			470*		
**Year of entry**						
2017–18	338 (74.6)			438 (93.2)		
2018–19	115 (25.4)			32 (6.8)		
**Age (years)**						
provided age information	449 (99.1)	19.19 (2.72)	17–40	384 (81.7)	18.90 (1.85)	17–39
did not provide information	4 (0.9)			86 (18.3)		
**Gender**						
Female	205 (45.3)			195 (41.5)		
Male	216 (47.7)			155 (33.0)		
Other (did not provide information/declared)	32 (7.0)			120 (25.5)		
**First generation go to university**						
Yes	230 (50.8)			175 (37.2)		
No	211 (46.6)			204 (43.4)		
Did not provide information	12 (2.6)			91 (19.4)		
**Ethnicity**						
White	313 (69.1)			337 (71.7)		
BAME	140 (30.9)			102 (21.7)		
did not provide information	n/a			31 (6.6)		
**Discipline**						
Business	199 (43.9)			112 (19.2)		
Law	98 (21.6)			94 (16.2)		
Biological sciences	39 (8.6)			27 (4.6)		
Geology	63 (13.9)			74 (12.7)		
Film	41 (9.1)			61 (10.5)		
Music	13 (2.9)			8 (1.4)		
History	N/A			206 (35.4)		

*In sample two, demographic information was only available for 470 participants (i.e., non-business participants). Details on year of entry, age, gender, first generation to go to university, and ethnicity were based on these 470 participants.

Sample one using pre-intervention data comprised 453 first year undergraduate students from across a range of disciplines. There were 205 (45.3%) females, 216 (47.7%) males, and 32 (7%) not reporting their gender. Ranging in age 17 to 40 years (Mean = 19.19, SD = 2.72), slightly over two thirds of participants (69.1%) identified themselves as White. Two hundred and thirty participants (50.8%) were the first person in their immediate family to study at a university.

Sample two using post-intervention data, comprised 582 first year students across a similar range of disciplines. Within this data set, of the 470 that provided full demographic data, 195 (41.5%) identified as females, 155 (33%) as males, and 120 (25.5%) providing no such information. Ranging in age 17–39 years (Mean = 18.90, SD = 1.85), the majority of those 470 participants (71.7%) identified themselves as White, with 175 participants identifying as the first person in their immediate family to study at a university. After addressing missing data, an overlap of 31 participants was identified between the pre-and post- intervention samples. Mindful of the issue of overfitting (Fokkema and Greiff, 2017), the overlapping data was removed when performing confirmatory factor analysis.

Demographic information was collected using a questionnaire requesting information on year of entry, gender, ethnicity, age, discipline, focus of study and whether the students were the first in their family to attend university. Outcome data included students’ end of module marks which were calibrated from the number and weighting of assessment tasks being summatively assessed within modules as part of a program of study.

Ethical approval for the collection and use of data was obtained from the University’s ethics committee in accordance with the institutional ethics policy and General Data Protection Requirements (GDPR). Students and lecturers had the right to withdraw consent to use their data at any time.

### 4.4. Instrument

**Figure 2:**
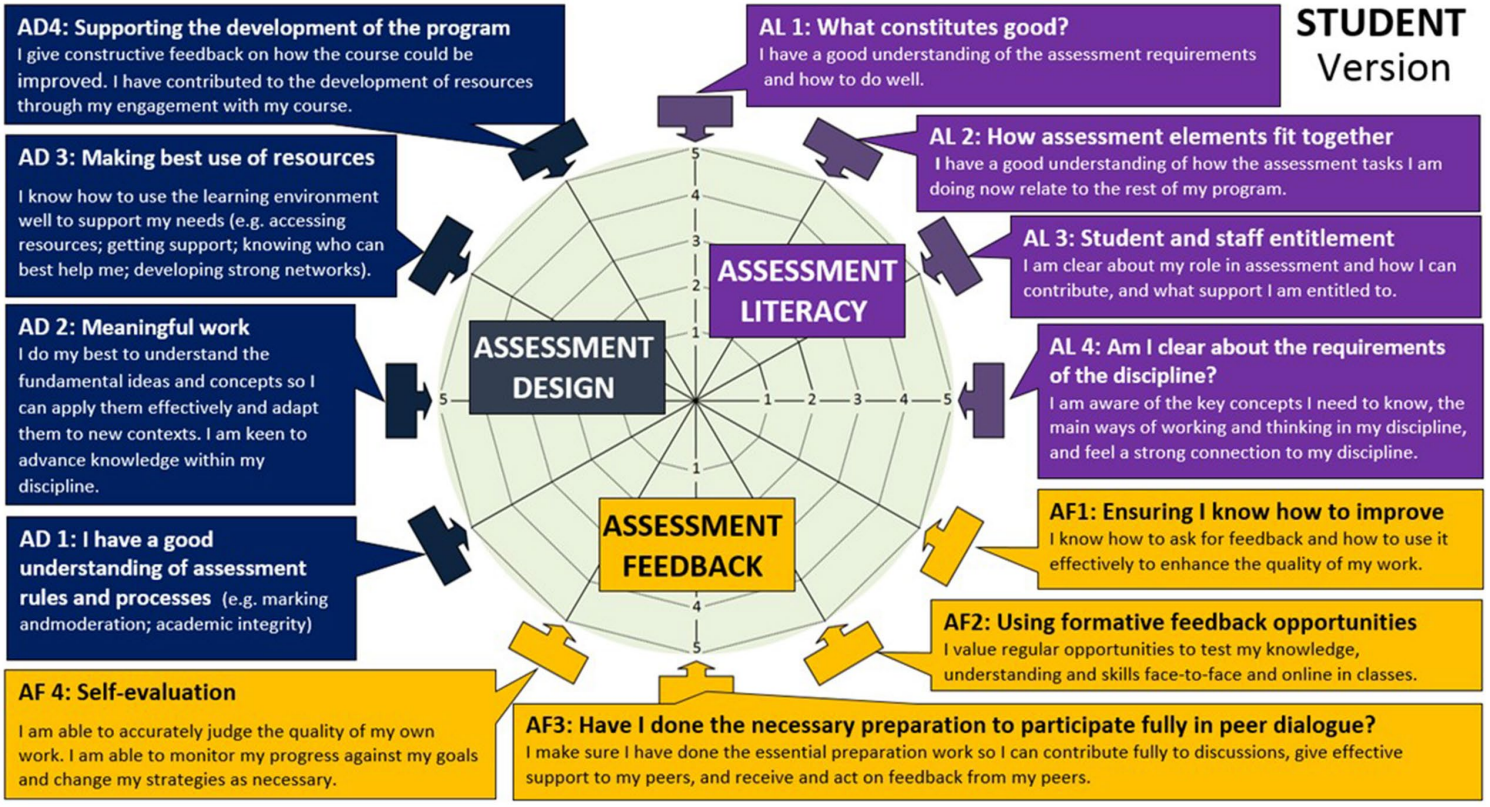
Assessment engagement questions—student version using the equity, agency, transparency framework (Evans, 2016, 2022).

The Assessment Engagement Scale (*AES*) comprises 12 items as depicted in Figure 2 and Table 2. The scale was derived from the Equity, Agency and Transparency Assessment (EAT) Framework (Evans, 2016, 2022). The instrument was designed to measure students’ perceptions of their engagement in assessment and feedback in relation to specific self-regulatory and agentic engagement behaviors. There are currently no instruments used in higher education that bring these constructs together to explore how students’ and academics’ navigate assessment within higher education.

**Table 2:** Descriptive statistics for 12 original Assessment Engagement Scale (AES) items.

AES item	M	SD	Skewness	Kurtosis
1. I have a good understanding of the assessment requirements, and how to do well	3.26	0.82	−0.42	0.53
2. I have a good understanding of how the assessment tasks I am doing now relate to the rest of my program	2.94	0.90	−0.29	0.24
3. I am clear about my role in assessment and how I can contribute, and what support I am entitled to	3.15	0.97	−0.30	−0.06
4. I am clear about the requirements of the discipline	3.40	0.85	−0.67	1.02
5. I know how to ask for feedback and use feedback effectively to enhance the quality of my work	3.41	0.84	−0.38	0.45
6. I value regular opportunities to test my knowledge, understanding and skills in class and online	3.15	0.93	−0.35	0.16
7. I make sure I have done the essential preparation work so I can contribute fully to discussions and give effective support to my peers	3.09	0.93	−0.14	0.20
8. I am able to accurately judge the quality of my own work	3.05	0.84	−0.32	0.54
9. I have a good understanding of assessment rules and processes (e.g., marking and moderation)	2.95	0.90	−0.32	0.19
10. I do my best to understand fundamental ideas and concepts so I can adapt and apply them to new contexts. I am keen to advance knowledge within my discipline	3.40	0.73	−0.60	1.46
11. I know how to use the learning environment well to support my needs (e.g., accessing resources; getting support; knowing who can best help me; developing strong networks)	3.32	0.83	−0.61	0.85
12. I give constructive feedback on how the course could be improved, and I have contributed to the development of resources through my engagement with the course	3.00	0.86	−0.20	0.37

The Supplementary Table S1 demonstrates the evolution of the Assessment Engagement Scale drawing on the EAT Framework’s dimensions and underpinning constructs, and the substantive literature base underpinning it as already noted in Section 3. A key aim of the Assessment Engagement Scale was to explore students’ perceptions of their confidence in understanding assessment requirements and ability to do well, linked to students’ self-regulatory skills including filtering capacity (Fyfe and Rittle-Johnson, 2016), cue consciousness (Van Merrienboer and de Bruin, 2019) and self-efficacy (Pintrich, 2004) (see Figure 1). Assessment literacy constructs such as understanding the requirements of assessment, recognizing the value of assessment to support learning, perceptions of ability to accurately judge the quality of one’s own work, and goals are captured (Smith et al., 2013), along with “*student savviness*” in how students agentically navigate the assessment feedback landscape to support their learning (Evans, 2013).

#### 4.4.1. Developing the assessment engagement scale

The nature and wording of the 12 scale items evolved through extensive engagement with academics, professional services teams, and students in naturalistic settings across a range of disciplines, contexts, and institutions. While the sample used to validate the student version of the instrument came from one UK Russell group university involving mainly undergraduates, students from across a wide range of disciplines (*n* = 48) were involved in the development of the scale. Furthermore, the scale had evolved in consultation with lecturers and students across many different types of institutions and countries.

Students were actively engaged in the development of the Assessment Engagement Scale as part of a third person perspective (Rosenfeld and Rosenfeld, 2011). The scale items were refined through lecturer (38 interviews) and student interviews (70 interviews involving approximately 300 students), and trialing with students and lecturers across faculties and disciplines (*n* = 48) at one UK Russell group university.

To ensure fidelity to the concepts and principles underpinning the Assessment Engagement Scale, “a vignette of statements” was developed for each scale item to provide a comprehensive picture of the items making up the survey to support dialog between lecturers and students as to the purposes of, and respective roles within assessment. In aiming to be parsimonious the statements were made as short and simple as possible while also aiming to capture the essential essence of the variable we were seeking to measure.

Students were asked to rate the 12 overarching statements at a minimum of two points in time (pre-and post-pedagogical interventions) using a five point Likert scale to assess their perceived levels of engagement with 1 representing “strongly disagree” and 5 “strongly agree” (Figure 1). Each of the scale items was deemed sufficient to create enough variance to examine the relationship between items and scales and ensure adequate coefficient alpha reliability estimates (Harris, 2008).

## 5. Data analysis

### 5.1. Establishing construct, criterion, and convergent validity

To explore the diagnostic potential of the Assessment Engagement Scale, its factor structure, and predictive validity, as a measure of criterion validity, and convergent validity were examined using pre-and post-test data student responses following pedagogical interventions to support student self-regulation of assessment (Evans et al., 2019). Three-phase statistical analyses on separate datasets collected at two points in time were undertaken using Hinkin et al. (1997) instrument development procedure to address our core research questions

- Objective 1: To explore the factor structure of the *AES*, an exploratory factor analysis (EFA) was used on sample one data (pre-intervention data) to explore the interrelationships among variables. Internal reliability was also examined. This was followed by a confirmatory factor analysis (CFA) using sample two (post-intervention data) to confirm the structure underlying the set of variables (Van Prooijen and Van Der Kloot, 2001).
- Objective 2: To ascertain predictive validity, as a measure of criterion validity, correlations between the *AES* scores and students’ end-of-module marks were analysed Using aggregated data, we further explored whether group differences existed before the implementation of the assessment interventions when *AES* scores were compared against a range of demographics and individual difference variables (e.g., gender, ethnicity, first in family to go to university), and in students’ module performance when the end-of-module marks were compared against the same demographic variables.
- Objective 3: The convergent validity of the *AES* was tested using an Assessment Literacy Survey (ALS) scale (Smith et al., 2013). The reliability of the 17-item ALS had previously been checked for use with a comparative sample of students (Zhu and Evans, 2022). While the *AES* aims to capture students’ approaches to assessment which extend beyond assessment literacy, we would expect a relationship between the two measures given that the 17-item ALS has four subscales including student understanding of assessment (assessment understanding, AU, 6 items), students’ use of assessment tasks to facilitate their learning (assessment for learning, AL, 5 items), students’ orientation to make minimum amount of effort in completing assessment tasks (tasks minimum effort orientation, MEO, 3 items), and the ability of students to judge the quality of their own and peers’ assessment work (assessment judgment, AJ, 3 items). As with the items in the *AES*, all items in the ALS were rated on a five point Likert scale ranging from 1 (strongly disagree) to 5 (strongly agree).
- Objective 4: The utility of the *AES* was ascertained from secondary data analysis of the outcomes of the self-regulatory assessment project reported on in Evans et al. (2018, 2019).

SPSS V.25 and AMOS V.25 were used to process and analyse the data, the former for descriptive analysis, the EFA, *t*-tests, analysis of correlations, and internal reliability, the later for the CFA. Before the EFA, missing value analysis of the 12 Assessment Engagement Scale items showed that the proportion of missing Assessment Engagement Scale values in sample one was 4% which was below the rule of thumb (5%) as suggested by Jakobsen et al. (2017), and that “missingness” appeared to be missing at random. Therefore, the expectation–maximization (EM) method (Dong and Peng, 2013) was applied to sample one to replace missing Assessment Engagement Scale data. Mindful that effects of small samples and missing data on EFA results remain inconclusive in literature, we applied the EM method to maximize sample size (McNeish, 2017). Listwise deletion was applied to deal with missing Assessment Engagement Scale values in sample two, resulting in a final sample of 185 participants for performing CFA.

### 5.2. Assessing the data and establishing the factor structure of the assessment engagement scale

To establish the dimensional structure and internal reliability of the Assessment Engagement Scale, exploratory factor analysis (EFA) was conducted on sample one using principal components analysis (PCA) (Pett et al., 2003). As part of initial data screening (Field, 2009), items were considered for elimination if (i) absolute skew values were >2.0 and absolute kurtosis values <7.0 (Kim, 2013), (ii) a large number of relatively low inter-item correlations (below 0.30), (iii) very high inter-item correlations suggesting multicollinearity (above 0.90), and (iv) low inter-total correlations (below 0.30).

Oblique rotation was applied as we expected the dimensions underlying the Assessment Engagement Scale to be inter-correlated. EFA results were examined with respect to sampling adequacy for analysis (Kaiser-Meyer-Olkin value >the acceptable limit of 0.50), item grouping (Bartlett’s test of sphericity *p* < 0.05), diagonals of anti-image correlation matrix (above 0.50), scree plots, eigenvalues (over Kaiser’s criterion of 1), and the percentage of variance explained (Costello and Osborne, 2005; Field, 2009). Further item elimination was carried out where items demonstrated: low communalities (less than 0.30), low individual item loadings onto exacted factors (below 0.35), or complex cross-loadings on two extracted factors without a difference of 0.30 or above between loadings on the primary and the other factors.

Internal reliability was analysed using Cronbach’s Alpha (*α*), with values >0.70 preferred given the exploratory purpose (Tavakol and Dennick, 2011; Taber, 2018). Predictive validity was assessed using Pearson’s correlation coefficient (*r*). Correlations between the overall and subscale pre-intervention Assessment Engagement Scale scores and students’ learning outcomes were investigated. The independent *t*-test was used to analyse group differences in these scores.

CFA was conducted on sample two using maximum likelihood solution to assess whether the EFA-generated structure of the Assessment Engagement Scale could be supported. To evaluate CFA results we used a combination of model fit indices (Hu and Bentler, 1995; Hinkin et al., 1997). A range of model fit indices and thresholds were examined (e.g., Hu and Bentler, 1995; Kline, 2005; Schreiber et al., 2006; Kline, 2016; Whittaker, 2016), namely: non-significant chi-squares (*χ*^2^) values, *χ*^2^/df < 2.00, Tucker-Lewis Index (TLI) and comparative fit index (CFI) > 0.90, the root mean-square error of approximation (RMSEA) up to 0.08 and PCLOSE >0.05.

## 6. Results

### 6.1. Confirming the factors underpinning the Assessment Engagement Scale

Descriptive statistics for individual items of the Assessment Engagement Scale are presented in Table 2. The 12 Assessment Engagement Scale items entered the first EFA using data from 453 participants. This provided a ratio of approximately 38 participants per item which is larger than the ratio of 30 participants per item recommended to make factors stable (Yong and Pearce, 2013). No items were eliminated during preliminary data screening. The iterative process of EFA resulted in subsequent elimination of three complex items (items 2, 8, and 10) due to cross-loadings. The final EFA with the remaining nine items yielded a KMO value of 0.84 and a significant Bartlett’s test of sphericity [*χ*^2^ (36) = 1025.84, *p* < 0.001]. These results verified that sample one was suitable for factor analysis. Two components had eigenvalues over 1. Inspection of the scree plot confirmed this two-factor solution.

The final two-factor solution (shown in Table 3) accounted for 54.92% of the overall variance: factor 1 containing five items (38.58%) and factor 2 containing four items (16.34%). Anti-image diagonals for the remaining nine items were 0.62 and above, and item communalities after extraction ranged from 0.47 to 0.65 with an average of 0.55. Internal reliabilities for the two factors suggest each subscale as a reliable measure (*α* = 0.79 for factor one with item-total correlation ranging from 0.51 to 0.60; *α* = 0.72 for factor two with item-total correlation ranging from 0.49 to 0.55). According to the component correlation matrix there was a positive correlation between the two factors (*r* = 0.36) which would be expected given the highly interconnected nature of the constructs we are exploring. Considering the items loading on the two factors, factor one represents *Understanding of the Assessment Context* (UAC) including one’s role within it, and confidence in navigating assessment requirements, and factor two represents *Realizing Engagement Opportunities* (*REO*) (i.e., willingness to engage and ability to utilize the assessment context effectively to support one’s understanding).

**Table 3:** Summary of exploratory factor analysis results with Cronbach’s alpha (*n* = 453).

Rotated factor loadings		
AES item	Factor 1	Factor 2
3. I am clear about my role in assessment and how I can contribute, and what support I am entitled to	0.78	
5. I know how to ask for feedback and use feedback effectively to enhance the quality of my work	0.76	
1. I have a good understanding of the assessment requirements, and how to do well	0.76	
4. I am clear about the requirements of the discipline	0.68	
9. I have a good understanding of assessment rules and processes (e.g., marking and moderation)	0.67	
7. I make sure I have done the essential preparation work so I can contribute fully to discussions and give effective support to my peers		0.77
12. I give constructive feedback on how the course could be improved, and I have contributed to the development of resources through my engagement with the course		0.64
11. I know how to use the learning environment well to support my needs (e.g., accessing resources; getting support; knowing who can best help me; developing strong networks)		0.63
6. I value regular opportunities to test my knowledge, understanding and skills in class and online		0.70
% of variance explained	38.58	16.34
Internal reliability (Cronbach’s *α*)	0.79	0.72

Item loadings < 0.35 have been suppressed.

**Figure 3:**
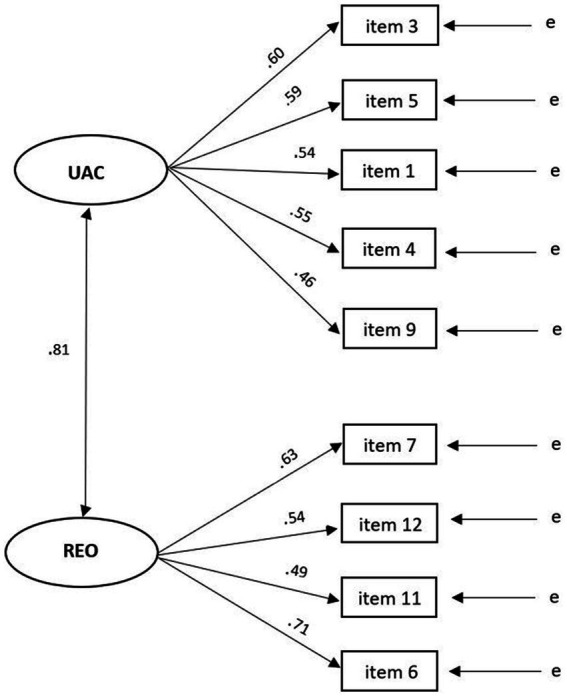
Standardized estimates for confirmatory factor analysis: two factor solution for the AES (N = 185, χ^2^ = 35.747, p > 0.05, df = 26, χ^2^/df = 1.375; TLI = 0.955, CFI = 0.967, RMSEA = 0.045; UAC, Understanding of Assessment of Context; REO, Realizing Engagement Opportunities; e, Error; *p* < 0.05 for all estimates).

The CFA confirmed the two-factor structure model with nine items generated by the EFA as shown in Figure 3. The overall chi-square was not statistically significant (*χ*^2^ = 35.747, *p* > 0.05, df = 26). Model fit indices used to assess the model’s overall goodness of fit were greater than conventional thresholds for an acceptable fit (*χ*^2^/df = 1.375, TLI = 0.955, CFI = 0.967, RMSEA = 0.045 which fall between 0.000 and 0.079, PCLOSE = 0.556). All item or factor loadings were statistically significant (*p*s < 0.05) (see Figure 3). Descriptive statistics for the final Assessment Engagement Scale, and its subscales are presented in Table 4.

**Table 4:** Summary of descriptive statistics for the AES after CFA (*n*_(listwise)_ = 185).

	No. of items	M	SD	Range	Skewness	Kurtosis
Overall AES	9	3.56	0.54	2.22–5.00	0.24	0.21
UAC	5	3.63	0.57	2.10–5.00	0.24	0.14
REO	4	3.40	0.69	1.75–5.00	0.23	−0.32

AES, assessment engagement scale; UAC, Understanding of Assessment Context; REO, Realizing Engagement Opportunities; M and SD represent mean and standard deviation.

### 6.2. Predictive validity: assessment engagement scale as an exploratory instrument

To examine and exemplify the diagnostic potential of the Assessment Engagement Scale as an exploratory instrument, students’ perceptions of engagement, their module mark as a measure of learning outcomes (Table 5), and group differences were analysed (Table 6).

**Table 5:** Pearson correlation: student learning outcome and *AES* scores (subscale and overall).

	Overall AES	UAC	REO	Module mark
Overall AES		0.76**	0.89**	0.16**
UAC			0.40**	0.20**
REO				0.10*

Module marks were from 339 students; AES, Assessment Engagement Scale; UAC, Understanding of Assessment Context; REO, Realizing Engagement Opportunities; *p < 0.05, **p < 0.01.

**Table 6:** Independent sample *t*-test results: *AES* scores (overall and subscale) and student learning outcome.

Demographic	Score	Groups	M	SD	SE	*t*	df	*P*_two-tailed_
Gender	Overall AES	Female	3.54	0.64	0.04	−0.34	419	0.73
		Male	3.56	0.59	0.04			
	UAC	Female	3.19	0.69	0.05	−1.21	419	0.23
		Male	3.26	0.59	0.04			
	REO	Female	3.18	0.66	0.05	0.22	419	0.83
		Male	3.16	0.63	0.04			
	Module mark	Female	62.08	13.51	1.12	0.92	307	0.36
		Male	60.57	15.19	1.19			
Ethnicity	Overall AES	White	3.49	0.62	0.04	−2.03	451	<0.05
		BAME	3.62	0.62	0.05			
	UAC	White	3.20	0.64	0.04	−1.73	451	0.09
		BAME	3.31	0.65	0.06			
	REO	White	3.10	0.66	0.04	−1.82	451	0.07
		BAME	3.22	0.65	0.05			
	Module mark	White	60.83	13.90	0.89	−1.13	337	0.26
		BAME	62.74	14.34	1.46			
First-generation university student	Overall AES	Yes	3.55	0.63	0.04	0.74	439	0.46
		No	3.51	0.61	0.04			
	UAC	Yes	3.27	0.66	0.04	1.34	439	0.18
		No	3.19	0.62	0.04			
	REO	Yes	3.15	0.67	0.04	0.44	439	0.66
		No	3.13	0.64	0.04			
	Module mark	Yes	61.81	15.45	1.19	0.64	328	0.52
		No	60.80	12.66	1.00			

AES, Assessment Engagement Scale; UAC, Understanding of Assessment Context; REO, Realizing Engagement Opportunities.

Students’ end-of-module marks were positively but weakly correlated to *pre-intervention Understanding of the Assessment Context* (*r* = 0.20, *p* < 0.01), *Realizing Engagement Opportunities* (*r* = 0.10, *p* < 0.05) and the pre-intervention overall Assessment Engagement Scale score (*r* = 0.16, *p* < 0.01).

Marginal differences were found between male and female students in pre-intervention subscale and overall Assessment Engagement Scale scores, and these gaps were not statistically significant at the 0.05 level [overall Assessment Engagement: *M*s = 3.56 and 3.54, *t*(419) = −0.34, *p* = 0.73; *Understanding of the Assessment Context*: *M*s = 3.26 and 3.19, *t*(419) = −1.21, *p* = 0.23; *Realizing Engagement Opportunities*: *M*s = 3.16 and 3.18, *t*(419) = 0.22, *p* = 0.83]. Students from Black, Asian and minority ethnic (BAME) groups were scored slightly but significantly higher than their white peers on overall *Assessment Engagement* scores before interventions [*M*s = 3.62 and 3.49 for BAME and White students respectively, *t*(451) = −2.03, *p* < 0.05]. BAME students also scored slightly higher on the two Assessment Engagement Scale subscales in comparison to white students, but the *p* values associated with these gaps were of marginal statistical significance (*p* = 0.09 for *Understanding of the Assessment Context*: and 0.07 for *Realizing Engagement Opportunities*). BAME students achieved higher marks than their white peers (*M*s = 62.74 and 60.83), although the differences in grades were not statistically significant.

### 6.3. Convergent validity: assessment engagement scale relationship to related measures

Statistically significant relationships were found between the 2 factors comprising the *AES: Understanding of the Assessment Context* (UAC) and *Realizing Engagement Opportunities* (REO) with Smith et al.’s (2013) Assessment Literacy Survey (ALS). Our findings, as outlined below, confirm the convergent validity of the *AES*. We would expect a relationship between the scales of the ALS and the AES given that one core element captured by the AES is students’ understanding of the requirements of assessment. We would also expect the relationships between the *REO* factor of the *AES* and the ALS to be weaker than that for the *UAC*, given the REO focuses more on student engagement but still requires an understanding of assessment requirements.

In summary, the directions of all correlations between the *Understanding of the Assessment Context (UAC)* and ALS scores (*n* = 124–131) were identified as anticipated: the *UAC* is positively and moderately correlated to Assessment Understanding (*r* = 0.49, *p* < 0.001), Assessment for Learning (*r* = 0.33, *p* < 0.001), and Assessment Judgment (*r* = 0.46, *p* < 0.001), while negatively and moderately correlated to Minimum Effort Orientation (*r* = −0.35, *p* < 0.001). Similarly, *Realizing Engagement Opportunities* (REO) significantly correlates to: AL (*r* = 0.30, *p* < 0.001); MEO (*r* = −0.30, *p* < 0.001), AU (*r* = 0.30, *p* < 0.001), and AJ (*r* = 0.24, *p* < 0.01). But these correlations are small, and weaker than those between scale 1 and assessment literacy.

### 6.4. The utility of the assessment engagement scale

Use of the Assessment Engagement Scale (*AES*) led to the adoption of more self-regulatory approaches to assessment design resulting in greater engagement with students. Engagement was dependent on building lecturer and student confidence in working in partnership with each other as reported in previous research (Evans et al., 2018, 2019). Use of the scale opened up discussion amongst lecturers regarding their beliefs about assessment and the student role in it, and enabled the development of shared understandings and increased consistency in addressing the basics of effective assessment practice across modules (Evans et al., 2019). In five of nine interventions, using matched data sets, students’ performance improved; students’ perceptions of their ability to judge the quality of their own work was noted in four of these. In six disciplines out of nine, positive relationships were found between perceptions of engagement and academic grades, although in one subject, perceptions of engagement were inversely related to performance. In-depth analysis revealed differences in the ways that different groups of students engaged over time (i.e., ethnicity, socio-economic status, first in family to attend university, gender) with variable impacts on performance. Data allowed comparison within and across modules and programs to identify shared patterns of engagement and also those unique to specific disciplines/modules.

## 7. Discussion

### 7.1. Confirming validity

Our analyses confirmed the reliability and validity of the Assessment Engagement Scale, providing empirical value to the theoretically-informed assessment framework (EAT) (Evans, 2016, 2022). Face validity of the scale was established through systematic mapping of the scale items to the research literature and extensive use of the measure in varied naturalistic settings (see Supplementary Table S1). Construct and criterion validity and reliability of the Assessment Engagement Scale were confirmed through exploratory and confirmatory factor analyses based on student data. The end product was a two-factor scale with nine items. Content validity and internal consistency reliability provided supportive evidence of construct validity. Providing further evidence of construct validity [i.e., examination of the extent to which the Assessment Engagement Scale correlated with measures designed to assess similar constructs (i.e., convergent validity) was also established through comparison of the *AES* to Smith et al.’s (2013) Assessment Literacy Survey].

### 7.2. Identification of underpinning constructs

Two underpinning constructs were identified: factor one representing *Understanding of the Assessment Context* (UAC) including five items, and factor two comprising *Realizing Engagement Opportunities* (REO) involving four items. Factor one integrates what we know about assessment literacy (understanding of the requirements of assessment and what good quality work looks like), to include knowership of process and context (including the tools of assessment such as assessment criteria, regulatory frameworks, knowledge of discipline, understanding of peer feedback), awareness of the potential of assessment to support learning, and confidence in one’s ability and role in navigating the requirements of assessment (Smith et al., 2013; Sadler, 2021). In focusing on intentionality and students’ conceptions of their role in assessment as actively involved in the construction of assessment meanings, this definition of assessment literacy is broader than existing definitions of the construct.

Factor two brings together notions of self-responsibility, and willingness to engage and contribute to assessment processes with understandings of how to use the environment to best effect to support learning (agentic engagement), including valuing working with others to enhance understanding for oneself. In this definition of engagement, students’ self-regulatory abilities are implicated in their ability to combine metacognitive, cognitive and affective dispositions in their management of their assessment environments. Together the two factors affirm the underpinning EAT Framework’s emphasis on the importance of designing learning environments that promote students’ agentic engagement with assessment, and ownership of the learning process. Students’ perceptions of their ability to take control of their learning impact their approaches to learning, and learning success (Ibarra-Saiz et al., 2020; Musso, 2020; Cervin-Ellqvist et al., 2021).

#### 7.2.1. Addressing excluded items

While the nine item solution is sound, there is need to further refine the Assessment Engagement Scale to address issues concerning the cross-loading of the three excluded items, which are key elements of effective assessment and feedback (Evans, 2013). These are complex constructs that draw on many interacting variables; it is important to unpack these further to refine the Scale. The findings also shine a spotlight on significant areas that need development within higher education assessment and feedback.

Scale item two (*I have a good understanding of how the assessment tasks I am doing now relate to the rest of my program*) relates to learners’ cognitive strategies, motivation and metacognitive actions, and we would expect this to impact engagement. If students are to be discerning in how they apply their efforts they need to know how assessment fits together (Schneider and Preckel, 2017). From a cognitive sciences perspective, many learners with wholistic preferences benefit from being able to see the bigger picture and how things are connected in order to focus on the task at hand (Kozhevnikov et al., 2014). However, understanding of the connections between different assessment elements of the taught program was problematic for lecturers and students reflecting a significant design issue within higher education assessment especially given the increasing complexity and choice in how programs are configured (Evans et al., 2019).

Scale item eight (*I am able to accurately judge the quality of my own work*) encompasses the ability to monitor progress in the moment and to judge the overall quality of one’s work; identified as fundamental in supporting learner success and independence in learning (Boud and Molloy, 2013; Tai et al., 2018). Self-evaluative capacity, identified as notoriously difficult to master (Eva and Regehr, 2013), requires opportunities for ongoing student engagement in activities that enable students to internalize standards for themselves (e.g., peer assessment; marking and moderating work; writing criteria and rubrics, constant comparison etc.) (Sadler, 2021; Nicol, 2022). Many of our students at the beginning of their higher education assessment journeys lacked confidence in their ability to judge the quality of their own work, finding assessment criteria obfuscate and lacking direct relevance to the tasks at hand, with insufficient opportunities within their program of study to test their understanding for themselves (Evans et al., 2019). Students who did less well had poor cue consciousness, in that they failed to recognize opportunities to support development of essential skills (Van Merrienboer and de Bruin, 2019), suggesting the need for clearer signposting. Further work is needed to refine this construct and its constituent parts. The key issue as mentioned above is that it draws on many different self-regulatory skills.

Scale item 10 (*I do my best to understand fundamental ideas and concepts so I can adapt and apply them to new contexts. I am keen to advance knowledge within my discipline*) aimed to capture students’ understanding of, and proclivity for a deep approach to learning (Asikainen and Gijbels, 2017). Students reported difficulties in identifying what the key concepts underpinning programs of study were (Evans et al., 2019). Lecturers and students alike had varied conceptions of what a deep approach in a specific subject was with significant impact on motivation and goals (McCune and Entwistle, 2011).

### 7.3. Enhancing engagement

The Assessment Engagement Scale proved powerful in promoting discussion of students’ and lecturers’ conceptions and beliefs about student engagement in assessment and development of shared values and goals (Price et al., 2011). Placing greater emphasis “at the front” of learning to support focused and aligned assessment goals is known to impact outcomes (Dent and Koenka, 2016).

Lecturers demonstrated increased criticality afforded by the Assessment Engagement Scale in their exploration of the extent to which curricula enabled students to actively engage with and take responsibility for their learning (Evans et al., 2018, 2019). Students found the Assessment Engagement Scale useful in focusing their attention on key elements of assessment, many of which they had not previously considered or had the confidence to have discussions about. Demonstrable impact on the quality of assessment design and student learning outcomes related to this institutional project is reported in Evans et al. (2019).

In seeking better understandings of individual differences, the Assessment Engagement Scale provides important information about student engagement and disengagement with assessment. The approach enabled timely interventions and meaningful staff-student dialogs around the impact of assessment design on the way in which students chose to, and were “enabled to engage with assessment” from individual and organizational perspectives (Vattøy et al., 2021).

### 7.4. Predictive potential

The Assessment Engagement Scale as a developmental and exploratory tool supports students’ learning transitions at critical points in their higher education journeys. The measure enables early identification, reflection, and discussion of conceptions of engagement and the role of lecturers and students in assessment. In focusing on process and outcomes, the Assessment Engagement Scale was used with students to explore changes in their levels of engagement with assessment and the impact of such behaviors on attainment. Use of the tool is particularly important during learning transitions and especially for those students whose self-regulation skills are weak, and who are, therefore, less likely to pick up assessment variation and read cues accurately. Using the Assessment Engagement Scale, enabled dynamic adjustments to be made to support students to make better use of their assessment contexts, develop the skills required and deploy them appropriately (Chipchase et al., 2017).

As a predictive tool, the Assessment Engagement Scale has considerable potential. Statistically significant relationships between Assessment Engagement Scale scores and student learning outcomes data were identified, with relatively strong and significant relationships being identified at module level (Evans et al., 2019). Student participation had a significant and direct effect on competence development (Ibarra-Saiz et al., 2020).

The Assessment Engagement Scale is concerned with the nature of such participation and what types of activities and strategies have most impact on learning. Using the Assessment Engagement Scale, individual and group engagement trajectories can be tracked to explore impacts of learning behaviors on outcomes, and to address learner misconceptions. Tracking perceptions of self-regulatory behaviors is important as they impact outcomes (Kyndt et al., 2011). In this research, perceptions of engagement were related to student outcomes but not in all cases (Evans et al., 2019), demonstrating the role of individual differences and context, and the need for thorough triangulation of data.

### 7.5. Areas for further development

#### 7.5.1. Measuring student engagement in assessment and feedback

The Assessment Engagement Scale measures students’ perceptions of engagement which may be different to their actual engagement in assessment. Poor self-regulators will struggle more than high self-regulators in accurately assessing the nature and quality of their contribution to the assessment process. Capturing students’ perceptions of engagement at an early stage and cross-referencing this with early baseline tests of competence is powerful in identifying learner profiles and targeting support accordingly.

Training was provided to students and academics in how to use the Assessment Engagement Scale as an integral element of curriculum delivery. Although all academics attended training and bespoke coaching events, the effectiveness of their approaches to supporting students’ self-reflection varied. Outcomes data provided rich information on what initiatives translated effectively across contexts, and also helped to identify misconceptions about assessment approaches which could be addressed in further training (Evans et al., 2019).

Different cohorts of students varied in their abilities to accurately report their engagement, and individual and organizational factors impacting this can be usefully explored. An inverse relationship between perceived student engagement and outcomes was noted in one of the nine assessment interventions. This finding is important in identifying where assessment initiatives are not landing as intended, and signaling the need to adapt strategy.

The Assessment Engagement Scale can be a very useful developmental tool for academics in working with students to explore the efficacy of their engagement in relation to ongoing formative and summative assessment tasks. The success of this process is dependent on building constructive assessment environments that enable learners (academics and students) to engage in dialog about the most effective ways to approach assessment that are also sensitive to individual student’s profiles.

#### 7.5.2. Unit of analysis

The relationships between students’ perceptions of engagement and learning outcomes were stronger when examined at the module level compared to aggregated data across modules and programs (Evans et al., 2019). These findings highlight the importance of context, and level of analysis considerations regarding the scale at which these variables are most meaningfully and usefully interrogated. Small changes in instruction at the module level can have large effects, and we argue that it is at this level of analysis that most useful information can be garnered to support student learning (Schneider and Preckel, 2017). To gain maximum benefit from the data, academics need training in how to use it to best effect.

#### 7.5.3. Scale and nature of enquiry

This article focuses on the development and application of the Assessment Engagement Scale in one specific context; replication is required across a range of contexts. However, our sample contained students from a wide range of disciplines, and the evolution of the underpinning theoretical framework and refinement of the *AES* scale was undertaken in collaboration with national and international partners supporting the face validity of the Assessment Engagement Scale.

The focus of this article was on the student version of the Assessment Engagement Scale, but further work is needed on the development and application of the lecturer version of the instrument, and across contexts to ascertain its potential to support effective professional development training in assessment, and to enhance the quality of assessment and feedback practices in higher education. Although not reported on in this article, there are significant learning opportunities for students to feedback on academics’ perceptions of the extent to which assessment contexts enable student engagement, and also for academics to feedback on their perceptions of student engagement in dialog with students to support shared understandings of what quality engagement looks like.

## 8. Conclusion

The Assessment Engagement Scale was developed to address the lack of accessible research-informed tools to promote understanding of high quality student and lecturer engagement in assessment; a key area of interest to higher education institutions globally. In drawing on best practice assessment principles (Evans, 2013), the Assessment Engagement Scale is unique in integrating research on effective assessment practice with that on self-regulated learning, agentic engagement, and individual differences in learning. Informed by a critical pedagogy (Waring and Evans, 2015) it supports lecturers in developing inclusive and participatory assessment environments within higher education. In doing so, it answers Dunlosky and Rawson’s (2019) call for translation of theories into authentic education settings.

This paper validates and confirms the underlying factor structure of the Assessment Engagement Scale. It is a reliable and valid tool that is suitable for use across contexts. The tool has capacity to identify students at risk, and to identify facilitators and barriers to access and engagement with assessment. From a utility perspective, the Assessment Engagement Scale is a unique and valuable bridging instrument in promoting student and lecturer engagement in assessment to support students’ self-regulatory skills development. It can bridge the teacher-student dialogic space in promoting shared understandings of meaningful engagement in assessment. The Assessment Engagement Scale, informed by a research-informed conceptual framework, places engagement at the center of curriculum thinking. In doing so, it gets to the heart of the matter in challenging lecturer and student beliefs about the role of students in the assessment process and provides a pragmatic route map for change.

As a predictive tool, the Assessment Engagement Scale can support students’ adoption of the most effective learning strategies, and identify students at risk of disengagement (Chipchase et al., 2017). The scale has demonstrated comprehensive enhancement in assessment design, reductions in differential learning outcomes for students, increased lecturer confidence in interacting in partnership with students, and career progression for lecturers and students (Evans et al., 2018, 2019). Lecturers’ in using the measure gained better understanding of students’ perspectives on assessment, enabling them to make manageable adjustments to assessment in real time, and in efficient ways.

In this paper we focused on the reliability and validity of the student version of the Assessment Engagement Scale. Further work is needed on refinement of scale items, and to ensure full coverage of the “engagement interface” (Trowler et al., 2021). Replication is required across wider contexts, but we are confident of the relevance and value of the scale in enhancing student and lecturer engagement in assessment given the preliminary findings, and engagement of key stakeholders across the sector in contributing to the evolution of it. There is considerable potential to explore the impact of lecturers’ conceptions of student engagement on the quality of assessment design and relationship to student learning outcomes.

Use of the scale across a wide range of contexts has highlighted key issues for higher education assessment practice in relation to attending to: (i) lecturer and student conceptions of and confidence in engaging in assessment and what this means (clarity around dimensions of engagement and why they are important); (ii) ensuring program coherence and signposting of how modules fit together; (iii) repeated opportunities for students to internalize what quality is for themselves, and (iv) clarification of what a deep approach to engagement looks like within disciplines.

Of critical importance is greater discernment around what quality engagement in assessment looks like within higher education, requiring deep understanding of the disciplinary context, individual differences, and the role of assessment in driving meaningful engagement. A key focus should be on how we generate the optimal conditions for engagement in assessment within higher education, which requires effective use of data and tools such as the Assessment Engagement Scale to open up discussions around the assessment process to support students in developing the skills they need now and in the future.

## Data availability statement

The raw data supporting the conclusions of this article will be made available by the authors, without undue reservation.

## Ethics statement

The studies involving human participants were reviewed and approved by the University of Southampton. The participants provided their written informed consent to participate in this study.

## Author contributions

All authors listed have made a substantial, direct, and intellectual contribution to the work and approved it for publication.

## Funding

This work was supported by funding from the Office for Students (OfS), United Kingdom, and the University of Southampton, Kingston University, and Surrey University through the “Maximising Student Success through the Development of Self-Regulation” project award led by CE (grant number L16).

## Conflict of interest

The authors declare that the research was conducted in the absence of any commercial or financial relationships that could be construed as a potential conflict of interest.

## Publisher’s note

All claims expressed in this article are solely those of the authors and do not necessarily represent those of their affiliated organizations, or those of the publisher, the editors and the reviewers. Any product that may be evaluated in this article, or claim that may be made by its manufacturer, is not guaranteed or endorsed by the publisher.
