# Supplementary material for: The development and validation of the assessment engagement scale
Source: Front Psychol. 2023 Jun 27;14:1136878. doi: 10.3389/fpsyg.2023.1136878 (PMC10333491; doi:10.3389/fpsyg.2023.1136878)
Supplement: Supplementary file 1 [file Data_Sheet_1.pdf]

Supplementary Material

The Development and Validation of the Assessment Engagement Scale

Carol Evans*, Xiaotong Zhu

*** Correspondence:** Carol Evans: evansc101@cardiff.ac.uk

Supplementary Table 1

frontiers

Supplementary Material TABLE 1: Development of Survey Items for the **Student** Assessment Engagement Scale

	EAT Framework constructs	Sources	*AES* first step - focus on understanding and engagement
AL 1	*To do well students need to know what they need to do and why, have a clear and accurate conception of what quality looks like, know how they are going to get there, and have the self-belief that they can.*		
	• Making assessment requirements and notions of quality explicit.<br>○ Importance of shared conceptions of quality between students and lecturers.<br>• Emphasis on beliefs and values.<br>• Importance of the role of individual differences in how learners make sense of and process information.<br>• Emphasis on development of quality and conditional use of self-regulation strategies.<br>○ Emphasis on activities to support student internalisation of standards.<br>○ Goal alignment.	Chan and Luo, 2021; Cumming and Van der Kleij, 2016<br>O'Donovan, 2017; Panadero and Alonso-Tapia, 2013<br>Ramaprasad, 1989; Sadler, 1989, 2010, 2013; Seifert, 2004<br>Friedlander et al., 2011; Fyfe and Rittle-Johnson, 2016; Prins et al., 2017<br>Dinsmore, 2017; Panadero, 2017<br>Fraile et al., 2017; Sadler, 2013, 2021<br>Farrell et al., 2017; Forsythe and Johnson, 2017; Lozano-Jiménez et al., 2021	Aligning goals, conceptions of quality and internalisation of standards:<br>*Do I know what constitutes good in assessment?*<br>↓<br>1. I have a good understanding of the assessment requirements, and how to do well.
AL 2	*Understanding how assessment fits together is essential to support effective learning and best use of resource.*		

	• Coherence and alignment of programme design.<br>○ Clear programme blueprints.<br>• Ensuring shared understandings of rationale underpinning programme design.<br>• Addressing information processing and cognitive styles in how information is understood.<br>• Self-regulatory planning skills/strategies – recognising connections between tasks and prioritising.	Biggs and Tang, 2015; Bearman et al., 2016; Crossley, 2014<br>El-Maaddawy and Deneen, 2017<br>Kozhevnikov et al., 2014; Dunlosky and Rawson, 2019<br>Dinsmore, 2017; Nguyen et al., 2017	Understanding the relationship of the parts to the whole impacting prioritisation of goals/tasks:<br>*Do I know how assessment elements fit together?*<br>↓<br>2. I have a good understanding of how the assessment tasks I am doing now relate to the rest of my programme.
AL3	*How students come to co-own their programs with lecturers and see themselves as active contributors to the assessment feedback process.*		
	• Lecturer expertise in promoting student engagement through design of assessment.<br>• Addressing student and lecturer beliefs and conceptions about the student role within assessment.<br>○ Training to support shared cultures of assessment.<br>• Students as co-partners in assessment (clarity of roles and expectations).<br>• Emphasis on promoting student engagement and co-ownership of assessment.	Boud and Molloy, 2013; Hawe and Dixon, 2017<br>ATN 2019; Formanek et al., 2017; Hawe and Dixon, 2017; Panadero et al., 2016<br>Boud and Molloy, 2013; Kumar et al., 2018; Matthews, 2019<br>Kuh et al., 2017; Montenegro, 2017; Reeve, 2013	Clarity around role(s) and expectations within assessment:<br>*Am I clear about my role/entitlement in assessment?*<br>↓<br>3. I am clear about my role in assessment and how I can contribute, and what support I am entitled to.

AL4	*Clarity around what it is to think, act and be within a discipline. How students and lecturers are inducted into a community of learners so that they feel that they can make a valued contribution to learning impacts progression and retention.*		
	• Clarifying what a deep approach within a discipline looks like.<br><br>• Importance of belonging and relatedness – relational capital in managing discipline interactions/transitions.<br><br>• Self-regulation training emphasis:<br>○ Signposting of core and threshold concepts.<br>○ Emphasis on epistemological understanding.<br>○ Modelling different ways of achieving excellence.	Clark 2012; Smyth et al., 2018; van Heerden et al., 2017<br><br>Bluic et al., 2011; Deci and Ryan, 2008; Van der Zanden et al., 2018<br><br>Meyer and Land, 2005<br><br>Feucht et al., 2017<br><br>Zimmerman, 2000	Understanding of what it is to be, act and be part of a discipline:<br><br>*Am I clear about the requirements of my discipline?*<br><br>↓<br><br>4. I am clear about the requirements of the discipline.
AF1	*Feedback is a finite resource that should be used judiciously and equitably. A focus on essentials is required to ensure students are able to access and act on the central feedback message.*		
	• Ensuring shared understandings of quality and efficiency of feedback.<br>○ Importance of simplifying the feedback message to promote accessibility – cognitive, metacognitive, and affective dimensions.<br>○ Importance of dialogue between lecturers and students to promote shared understanding of the purposes of feedback, and in relation to goals.	Henderson et al., 2019; Sadler, 2021<br><br>Van Merrienboer and de Briun, 2019; Friedlander et al., 2011<br><br>Eva and Regehr, 2013; Fyfe and Rittle-Johnson, 2016; van Heerden et al., 2017	Efficiency and accuracy in utilising, generating, and seeking feedback:<br><br>*Do I know how to ask/seek the right kind of feedback and how to improve my work from the feedback I receive?*

	• Importance of students' conceptions of feedback, personal beliefs, motives, schema, knowledge base, self-regulatory skills, assessment feedback history, and confidence.<br><br>• Feedback as co-constructed and multifaceted to include self-generation of feedback, seeking and utilisation of feedback cues from the environment.	Brown et al., 2016; Dawson et al., 2019; Forsythe and Johnson, 2017; Nash and Winstone, 2017; Waring and Evans, 2015<br><br>Ajjawi and Boud, 2017; Carless, 2020; Carless and Boud, 2018; Farrell et al., 2017; Nicol 2010, 2022	↓<br><br>5. I know how to ask for feedback and use feedback effectively to enhance the quality of my work.
AF2	*Early use of formative assessment opportunities assist students in understanding assessment requirements for themselves.*		
	• Understanding of individual differences in framing feedback opportunities – adaptive design.<br><br>• Providing early opportunities for students to calibrate judgements on the quality of their work, test preconceptions and schema, and support metacognitive monitoring accuracy.<br><br>○ Facilitating the progressive development of students' knowledge and skills – repeated opportunities to observe, emulate, test ideas.<br><br>○ Providing opportunities for repeated practice to support automation of skills drawing on information processing perspectives.	Fyfe and Rittle-Johnson, 2016; Tio et al., 2016<br><br>DiFrancesca et al., 2016; Paschold et al., 2014; Vermunt and Donche, 2017<br><br>Boud and Molloy 2013; DiFrancesca et al., 2016; Domenech et al., 2015; Sennhen-Kirchner et al., 2017<br><br>Domenech et al., 2015; Dunlosky et al., 2013; Panadero and Alonso-Tapia, 2013; Sennhenn-Kirchner et al., 2017	Engagement in learning activities to support understanding and generating feedback opportunities:<br><br>*Am I making full use of formative feedback opportunities?*<br><br>↓<br><br>6. I value regular opportunities to test my knowledge, understanding and skills in class and online.

AF3	*Students' contribution to the assessment process; engaging in the development of curriculum and peer support impacts students' evaluation capacity.*		
	• Promoting authentic peer engagement activities to support the self-assessment process, and co- and shared regulation.<br>○ Using peer assessment to promote self-assessment capabilities- understanding of quality for oneself.<br>○ Supporting individual agency and individual accountability within the peer assessment process.<br>• Developing students' filtering capacity - discernment in the selection and use of peer feedback.	Nicol et al., 2014; Rienties et al., 2019<br>Emke et al., 2017; Huitt et al., 2015<br>Ibarra-Saiz et al., 2020; Schneider and Preckel, 2017<br>Brown et al., 2016; Nicol and McCallum, 2021	Engagement in peer activities to support own and others' learning:<br>*Have I done the necessary preparation to participate fully in peer learning activities?*<br>↓<br>7. I make sure I have done the essential preparation work so I can contribute fully to discussions and give effective support to my peers.
AF4	*The quality of assessment design impacts student self-management of feedback processes.*		
	• Embedding the development of self-assessment skills throughout the curriculum.<br>• Student beliefs about self-assessment ability and emotional challenges of self-assessment.<br>• Promoting the development of self-monitoring and self-evaluation skills.<br>○ Working with students' to develop their cognitive abilities in aggregating perceptions over multiple experiences.	Boud et al., 2013; Di Francesco et al., 2016; Panadero and Alonso-Tapia, 2013; Panadero, 2017; Tai et al., 2018<br>Brown et al., 2016; DeNisi and Kluger, 2000; Driessen and Scheele, 2013<br>Eva and Regehr, 2011	Understanding of quality within a specific context and how to apply such knowledge to the evaluation of one's own work:<br>Monitoring and evaluation accuracy (critical reflection):<br>*Am I able to accurately assess my own work?*

	○ Modelling of self-assessment strategies to support accuracy and appropriate use of strategies.<br><br>○ Emphasis on immersing students in activities to promote internalisation of standards.	Zimmerman, 2000<br><br>Nicol and McCallum, 2021; Nicol, 2022; Sadler, 2021	↓<br><br>8. I am able to accurately judge the quality of my own work.
AD 1	*Ensuring robust and transparent processes and procedures with emphasis on QA literacy promotes ownership, access, and perceptions of fairness.*		
	• Ensuring transparency of policy and process.<br><br>• Clear benchmarking of standards and opportunities for students to work with lecturers in marking and moderation activities.<br><br>• Understanding of cognitive, political, social and cultural capital implicated in navigating the rules of assessment.<br><br>• Making local assessment cultures explicit- understanding of individual differences in how assessment rules are interpreted and enacted.<br><br>• Encouragement to challenge understandings of rules as part of an agentic approach.<br><br>• Providing opportunities for students to moderate and assess work and engage in discussions around how grading of work is decided.	Balloo et al., 2018; Dawson and Henderson, 2017<br><br>Gonsalvez et al., 2017; Sadler, 2016<br><br>James, 2014; Yee, 2016<br><br>Blasco, 2015; Grainger et al., 2017; Sadler, 2017<br><br>Montenegro, 2017; Reeve, 2013<br><br>Nicol, 2022; Sadler, 2021	Understanding of the rules of assessment – political literacy – how things are done:<br><br>*Do I have a good understanding of higher education assessment rules and processes*<br><br>↓<br><br>9. I have a good understanding of assessment rules and processes (e.g., marking and moderation).

AD2	*Importance of co-construction of the curriculum –cultivating a shared understanding of a deep approach and emphasizing the importance of authentic outputs- assessments that have value to the individual and community beyond the immediate assessment task.*		
	• Promotion of holistic and deep approaches to learning. Modelling of deep approaches and signposting key skills.	Asikainen and Gijbels, 2017; Dall'Alba and Barnacle, 2007; Riddell, 2015	Knowledge of the disciplinary conventions and what it is to be competent as a student of a specific discipline:
	• Emphasis on authentic practice and student ownership of assessment design, and development of useful products.	Ashford-Rowe et al., 2014; Nicol et al., 2014	Willingness to engage; sensitivity to context needs and individual responsibility in development of knowledge and understanding
	• Focus on process and not just product – progressive development of knowledge and skills throughout a programme.	Bearman et al., 2016; De Hertogh, 2014; Rienties and Toetenel, 2016	*Am I adopting a deep approach to my work in trying my best to understand for myself?*
	• Promotion of high level self-regulatory skills and efficiency in learning.	Dinsmore, 2017; Schneider and Preckel, 2017	↓<br>10. I do my best to understand fundamental ideas and concepts so I can adapt and apply them to new contexts. I am keen to advance knowledge within my discipline.

AD3	*Inclusive assessment promotes equal access to the curriculum and equal opportunities to do well.*		
	• Participatory pedagogy emphasising equal access to assessment and opportunities to do well.<br>• Emphasis on promoting agency and autonomy within the learning context including students' agentic engagement with assessment.<br>• Understanding of how individuals/groups make sense of information.<br>• Emphasis on cognitive, metacognitive and affective dimensions of learning (e.g., reducing cognitive load, teaching metacognitive skills, addressing confidence and motivations to include self-efficacy).	Rogers-Shaw et al., 2018; Scott et al., 2014<br>Deci and Ryan, 2008; Freire, 1996; Giroux, 2009<br>Kozhevnikov et al., 2014; Vermunt and Donche, 2017<br>Friedlander et al., 2011; Waring and Evans, 2015	Engagement in network development, feedback seeking, resource usage:<br>*Am I making best use of resources available?*<br>11. I know how to use the learning environment well to support my needs (e.g. accessing resources; getting support; knowing who can best help me; developing strong networks).
AD4	*Evaluation embedded within assessment design to ensure curriculum is the joint responsibility of lecturers and students.*		
	• Co-ownership/co-construction - the joint responsibility of lecturers and students in assessment design – importance of student voice as part of team-based design.<br>• Curriculum seen as dynamic.<br>o Informed use of data to support iterative development of it.<br>• Predictive use of data to ensure individual/group differences supported.<br>• Importance of self-regulation training for lecturers and students – exploring student and lecturer learning and teaching profiles.	Goldman et al., 2012; Mc Evoy, 2017; Smyth et al., 2018<br>Macfadyen et al., 2014; Mora et al., 2016; Nguyen et al., 2017; Whitworth and Wright, 2015; Youssef, 2017<br>Emke et al., 2017; Larrabee Sonderlund et al., 2019; McEvoy, 2017; Musso, 2020<br>Dorrenbacher and Perels, 2016; Fernández Ruiz et al., 2021	Sees importance of engagement in course development as an active contributor. Demonstrates awareness of impact of assessment design from own and others' perspectives:<br>*Am I giving useful feedback on how to enhance assessment feedback practice?*<br>12. I give constructive feedback on how the course could be improved, and I have contributed to the development of resources through my engagement with the course.

Additional References

Ajjawi, R., and Boud, D. (2017). Researching feedback dialogue: an interactional analysis approach. *Assessment & Evaluation in Higher Education* 42 (2), 252-265. doi:10.1080/02602938.2015.1102863

Ashford-Rowe, K., Herrington, J., and Brown, C. (2014). Establishing the critical elements that determine authentic assessment. *Assessment & Evaluation in Higher Education* 39 (2), 205-222. doi: 10.1080/02602938.2013.819566

ATN. (2019). ATN joint statement on authentic assessment. Australian Technology Network of Universities. *Australian Technology Network of Australian Universities* https://www.atn.edu.au/news-and-events/latest-news/atn-joint-statement-on-authentic-assessment/

Balloo, K., Evans, C., Hughes, A., Zhu, X., and Winstone, N. (2018). Explicit assessment criteria as the antithesis of 'spoon-feeding': how transparency in the assessment process can support students' self-regulatory development. *Frontiers in Education* 3. doi:10.3389/feduc.2018.00069

Bearman, M., Dawson, P., Boud, D., Bennett, S., Hall, M., and. Molloy, E. (2016). Support for assessment practice: developing the assessment design decisions framework. *Teaching in Higher Education* 21 (5), 545-556. doi: 10.1080/13562517.2016.1160217

Biggs, J., and Tang, C. (2015). Constructive alignment: an outcomes-based approach to teaching anatomy. In *Teaching Anatomy* (pp. 31-38). Springer. doi: 10.1007/978-3-319-08930-0_4

Blasco, M. (2015). Making the tacit explicit: rethinking culturally inclusive pedagogy in international student academic adaptation. *Pedagogy, Culture & Society* 23 (1), 85–106. doi: 10.1080/14681366.2014.922120

Bliuc, A.-M., Ellis, R. A., Goodyear, P., and Hendres, D. M. (2011). Understanding student learning in context: Relationships between university students' social identity, approaches to learning, and academic performance. *European Journal of Psychology of Education* 26, 417–433. doi: 10.1007/s10212-011-0065-6

Boud, D., Lawson, R., and Thompson, D.G. (2013). Does student engagement in self-assessment calibrate their judgement over time? *Assessment and Evaluation in Higher Education* 38 (8), 941-956. doi: 10.1080/02602938.2013.769198

Brown, G. T. L., Peterson, E. R., and Yao, E. S. (2016). Student conceptions of feedback: impact on self-regulation, self-efficacy, and academic achievement. *British Journal of Educational Psychology* 86 (4), 606–629. doi: 10.1111/bjep.12126

Carless, D. (2020). Longitudinal perspectives on students' experiences of feedback: a need for teacher–student partnerships. *Higher Education Research & Development* 39 (3), 425–438. doi: 10.1080/07294360.2019.168445

Carless, D., and Boud, D. (2018). The development of student feedback literacy: enabling uptake of feedback. *Assessment & Evaluation in Higher Education* 43 (8), 1315-1325. doi:10.1080/02602938.2018.1463354

Chan, C.K.Y., and Luo, J. (2021). A four-dimensional conceptual framework for student assessment literacy in holistic competency development. *Assessment & Evaluation in Higher Education* 46 (3), 451-466. doi: 10.1080/02602938.2020.1777388

Clark, I. (2012). Formative assessment: assessment is for self-regulated learning. *Educational Psychology Review* 24 (2), 205-249. doi: 10.1007/s10648-011-9191-6

Crossley, J.G. (2014). Addressing learner disorientation: give them a roadmap. *Medical Teacher* 36 (8), 685-691. doi: 10.3109/0142159X.2014.889813

Cumming J.J., and Van der Kleij, F.M. (2016). Effective enactment of assessment for learning and student diversity in Australia. In: Laveault D., & Allal L. (Eds.) *Assessment for L Learning: Meeting the Challenge of Implementation. The Enabling Power of Assessment*, vol 4. Springer, doi: 10.1007/978-3-319-39211-0_4

Dall'Alba, G., and Barnacle, R. (2007). An ontological turn for higher education. *Studies in Higher Education* 32 (6), 679-691. doi: 10.1080/03075070701685130

Dawson, P., and Henderson. M. (2017). How does technology enable scaling up assessment for learning? In D. Carless, S. Bridges, C. K. Y. Chan, & R. Glofcheski (Eds.), *Scaling Up Assessment for Learning in Higher Education* (pp. 209-222). Springer. doi: 10.1007/978-981-10-3045-1_14.

De Hertogh, L. B. (2014). Toward a revised assessment model: rationales and strategies for assessing students' technological authorship. *Composition Forum* 30, 13.

Deci, E. L., and Ryan, R. M. (2008). Self-determination theory: A macrotheory of human motivation, development, and health. *Canadian Psychology/Psychologie canadienne,* 49 (3), 182–185. doi: 10.1037/a0012801

DeNisi, A. S., and Kluger, A. N. (2000). Feedback effectiveness: can 360-degree appraisals be improved? *Academy of Management Executive* 14 (1): 129–139. doi: 10.5465/AME.2000.2909845

DiFrancesca, D., Nietfeld, J. L., and Cao, L. (2016). A comparison of high and low achieving students on self-regulated learning variables. *Learning and Individual Differences* 45, 228–236. doi: 10.1016/j.lindif.2015.11.010

Domenech, J., Blazquez, D., De la Poza, E., and Muñoz-Miquel, A. (2015). Exploring the impact of cumulative testing on academic performance of undergraduate students in Spain. *Educational Assessment, Evaluation and Accountability* 27 (2), 153-169. doi: 10.1007/s11092-014-9208-z

Driessen, E., and Scheele, F. (2013). What is wrong with assessment in postgraduate training? lessons from clinical practice and educational research. *Medical Teacher* 35 (7), 569-574. doi: 10.3109/0142159X.2013.798403

El-Maaddawy, T., and Deneen, C. C. (2017). Outcomes-based assessment and learning: trialling change in a postgraduate civil engineering course. *Journal of University Teaching and Learning Practice* 14 (1), article 10.

Emke, A.R., Cheng, S., Chen, L., Tian, D., and Dufault, C. (2017). A novel approach to assessing professionalism in preclinical medical students using multisource feedback through paired self- and peer evaluations. *Teaching and Learning in Medicine* 29 (4), 402-410. doi: 10.1080/10401334.2017.1306446

Farrell, L., Bourgeois-Law, G., Ajjawi, R., and. Regehr, G. (2017). An autoethnographic exploration of the use of goal-oriented feedback to enhance brief clinical teaching encounters. *Advances in Health Sciences Education* 22 (1), 91-104. doi: 10.1007/s10459-016-9686-5

Fernández Ruiz, J., Panadero, E., García- Pérez, D., and Pinedo, L. (2021). Assessment design decisions in practice: profile identification in approaches to assessment design. *Assessment and Evaluation in Higher Education,* 1–16. doi: 10.1080/02602938.2021.1937512

Feucht, F., Brownless, J. M. L., and Schraw, G. (2017). Moving Beyond Reflection: Reflexivity and Epistemic Cognition in Teaching and Teacher Education. *Educational Psychologist* 52 (4), 234-241. doi: 10.1080/00461520.2017.1350180

Formanek, M., Wenger, M. C., Buxner, S. R., Impey, C. D., and Sonam, T. (2017). Insights about large-scale online peer assessment from an analysis of an astronomy MOOC. *Computers & Education* 113, 243-262. doi: 10.1016/j.compedu.2017.05.019

Forsythe, A., and Johnson, S. (2017). Thanks, but no-thanks for the feedback. *Assessment & Evaluation in Higher Education* 42 (6), 850-859. doi: 10.1080/02602938.2016.1202190

Fraile, J., Panadero, E., and Pardo, R. (2017). Co-creating rubrics: the effects on self-regulated learning, self-efficacy and performance of establishing assessment criteria with students. *Studies in Educational Evaluation* 53, 69-76. doi: 10.1016/j.stueduc.2017.03.003

Freire, P. (1996) [1970]. *Pedagogy of the Oppressed*. Translated by M. B Ramos, Penguin.

Giroux, H. A. (2009). Critical theory and educational practice. In A. Darder, M. Baltodano and R. D. Torres (Eds.), *The Critical Pedagogy Reader (*pp. 27-51). New York, NY, Routledge.

Goldman, E., Swayze, F., Swinehart, S. S., Schroth, S. E. and. Scott, W. (2012). Effective curricular change through comprehensive course assessment using structure and processes to change outcomes. *Academic Medicine* 87 (3), 300-307. doi: 10.1097/ACM.0b013e318244739c

Gonsalvez, C.J., Wahnon, T., and Deane, F. P. (2017). Goal-setting, feedback, and assessment practices reported by Australian clinical supervisors. *Australian Psychologist* 52 (1), 21-30. doi: 10.1111/ap.12175

Grainger, P., Christie, M., Thomas, G., Dole, S., Heck, D., Marshman, M., and Carey, M. (2017). Improving the quality of assessment by using a community of practice to explore the optimal construction of assessment rubrics. *Reflective Practice* 18 (3), 410-422. doi: 10.1080/14623943.2017.1295931

Hawe, E., and Dixon, H. (2017). Assessment for learning: a catalyst for student self-regulation. *Assessment & Evaluation in Higher Education* 42 (8), 1181-1192. doi: 10.1080/02602938.2016.1236360

Henderson, M., Ajjawi, R., Boud, D., and Molloy, E. (2019).Identifying feedback that has impact. In Michael Henderson, R. Ajjawi, D. Boud, & E. Molloy (Eds.), *The Impact of Feedback in Higher Education* (pp. 15–34). Palgrave Macmillan. doi: 10.1007/978-3-030-25112-3_2

Huiit, T. W., Killins, A., and Brooks, W. S. (2015). Team-based learning in the gross anatomy laboratory improves academic performance and students' attitudes toward teamwork. *Anatomical Sciences Education* 8 (2), 95-103. doi: 10.1002/ase.1460

Kuh, G., O'Donnell, K., and Schneider, C. J. (2017). *HIPs at ten. Change: The Magazine of Higher Learning* 49 (5): 8-16. doi: 10.1080/00091383.2017.1366805

Kumar, R., Zusho, A., and Bondie, R. (2018). Weaving cultural relevance and achievement motivation into inclusive classroom cultures. *Educational Psychologist* 53 (2), 78–96. doi: 10.1080/00461520.2018.1432361

Larrabee Sønderlund, A., Hughes, E., and Smith, J. (2019). The efficacy of learning analytics interventions in higher education: a systematic review. *British Journal of Educational Technology 50* (5), 2594-2618. doi: 10.1111/bjet.12720

Lozano-Jiménez J.E., Huéscar, E., and Moreno-Murcia, J. A. (2021). From autonomy support and grit to satisfaction with life through self-determined motivation and group cohesion in higher education. *Frontiers Psychol.* doi: 10.3389/fpsyg.2020.579492.

Macfadyen, L.P., Dawson, S., Pardo, A., and Gasevic, D. (2014). Embracing big data in complex educational systems: the learning analytics imperative and the policy challenge. *Research & Practice in Assessment* 9, 17-28. http://www.rpajournal.com/dev/wp-content/uploads/2014/10/A2.pdf

Matthews, K. E. (2019). Rethinking the problem of faculty resistance to engaging with students as partners in learning and teaching in higher education. *International Journal for the Scholarship of Teaching and Learning* 13 (2), article 2. https://files.eric.ed.gov/fulltext/EJ1218288.pdf

McEvoy, J.P. (2017). Interactive problem-solving sessions in an introductory bioscience course engaged students and gave them feedback, but did not increase their exam scores. *FEMS microbiology letters* 364 (18). doi: 10.1093/femsle/fnx182

Meyer, J. H. F., and Land, R. (2005). Threshold concepts and troublesome knowledge (2): epistemological considerations and a conceptual framework for teaching and learning. *Higher Education* 49, 373-388.

Montenegro, A. (2017). Understanding the concept of student agentic engagement for learning. *Colombian Applied Linguistics Journal* 19 (1), 117–128. doi: 10.14482/calj.v19n1.10472

Mora, N., Caballe, S., and Daradoumis, T. (2016). Providing a multi-fold assessment framework to virtualized collaborative learning in support for engineering education. *International Journal of Emerging Technologies in Learning* 11 (7).

Nash, R. A., and Winstone, N. (2017). Responsibility sharing in the giving and receiving of feedback. *Frontiers in Psychology.* doi: 10.3389/fpsyg.2017.01519

Nicol, D. (2010). From monologue to dialogue: improving written feedback processes in mass higher education. *Assessment and Evaluation in Higher Education* 35, 501-517. doi: 10.108-/02602931002786559

Nicol, D., and McCallum, S. (2021). Making internal feedback explicit: exploiting the multiple comparisons that occur during peer review. *Assessment and Evaluation in Higher Education:* 1–19. doi: 10.1080/02602938.2021.1924620

Nicol, D., Thomson, A., and Breslin, C. (2014). Rethinking feedback practices in higher education: a peer review perspective. *Assessment & Evaluation in Higher Education* 39 (1), 102-122. doi: 10.1080/02602938.2013.795518

Nguyen, Q., Rienties, R., Toetenel, L., Ferguson, R., and Whitelock, D. (2017). Examining the designs of computer-based assessment and its impact on student engagement, satisfaction, and pass rates. *Computers in Human Behavior* 76, 703-714. doi: 10.1016/j.chb.2017.03.028

O'Donovan, B. (2017). How students beliefs about knowledge and knowing influence their satisfaction with assessment and feedback. *Higher Education* 74 (4), 617-633. doi: 10.1007/s10734-016-0068-y

Panadero, E., and Alonso-Tapia, J. (2013). Self-assessment: theoretical and practical connotations. when it happens, how is it acquired and what to do to develop it in our students. *Electronic Journal of Research in Educational Psychology* 11 (2), 551–576. doi: 10.14204/ejrep.30.12200

Panadero, E., Brown, G.T., and Strijbos, J. (2016). The future of student self-assessment: a review of known unknowns and potential directions. *Educational Psychology Review* 28, 803-830. doi: 10.1007/s10648-015-9350-2

Paschold, M., Huber, T., Zeissig, S. R., H. Lang, H., and Kneist, W. (2014). Tailored instructor feedback leads to more effective virtual-reality laparoscopic training. *Surgical Endoscopy* 28, 967-973.

Prins, F. J., de Kleijn, R., and Tartwijk, J. V. (2017). Students' use of a rubric for research theses. *Assessment and Evaluation in Higher Education* 42 (1), 128-150. doi: 10.1080/02602938.2015.1085954

Ramaprasad, A. (1983). On the definition of feedback. *Behavioral Science*, 28(1), 4-13. doi: 10.1002/bs.3830280103

Riddell, J. (2015). Performance, feedback, and revision: metacognitive approaches to undergraduate essay writing. *Collected Essays on Learning and Teaching* 8, 79-96.

Rienties, B., Tempelaar, D. T., Nguyen, Q., and Littlejohn, A. (2019). Unpacking the intertemporal impact of self-regulation in a blended mathematics environment. *Computers in Human Behavior* 100 (November 2019):,345-357. doi: 10.1016/j.chb.2019.07.007

Rienties, B., and Toetenel. L. (2016). The impact of learning design on student behaviour, satisfaction and performance: a cross-institutional comparison across 151 modules. *Computers in Human Behavior* 60, 333-341. doi: 10.1016/j.chb.2016.02.074

Rogers-Shaw, C., Carr-Chellman, D. J., and Choi, J. (2018). Universal design for learning: guidelines for accessible online instruction. *Adult Learning* 29 (1): 20-31. doi: 10.1177/104515951773550

Sadler, D. R. (2010). Beyond feedback: developing student capability in complex appraisal. *Assessment and Evaluation in Higher Education* 35, 535–550. doi: 10.1080/02602930903541015

Sadler, D. R. (2013). Opening up feedback: teaching learners to see. In S. Merry, M. Price, D. Carless, & M. Taras (Eds.), *Reconceptualising Feedback in Higher Education: Developing Dialogue with Students* (pp. 54–63). London, UK, Routledge.

Sadler, D. R. (2016). Three in-course assessment reforms to improve higher education learning outcomes. *Assessment & Evaluation in Higher Education* 41(7), 1081-1099. doi: 10.1080/02602938.2015.1064858

Sadler, D. R. (2017). Academic achievement standards and quality assurance. *Quality in Higher Education* 23 (2), 81–99. doi: 10.1080/13538322.2017.1356614

Seifert, T. (2004). Understanding student motivation. *Educational Research* 46 (2), 137-149. doi: 10.1080/0013188042000222421

Sennhenn-Kirchner, S., Goerlich, Y., Kirchner, B., Notbohm, M., Schiekirka, S., Simmenroth, A., and Raupach, T. (2017).The effect of repeated testing vs repeated practice on skills learning in undergraduate dental education. *European Journal of Dental Education* 22 (1), e42-e47. doi: 10.1111/eje.12254

Smyth, L., Chandra, V., and Mavor, K. I. (2018). Social identification and normative conflict: when student and educator learning norms collide. *Journal of Applied Psychology* 48 (6), 293-303. doi: 10.1111/jasp.12505

Tio, R., Stegmann, M., Koerts, J., van Os, T. W. D. P., and Cohen-Schotanus, J. (2016). Weak self-directed learning skills hamper performance in cumulative assessment. *Medical Teacher* 38 (4), 421-423. doi: 10.3109/0142159X.2015.1132411

van der Zanden, P. J. A. C., Denessen, E., Cillessen, A. H. N., and Meijer, P. C. (2019). Patterns of success: first-year student success in multiple domains. *Studies in Higher Education* 44 (11), 2081-2095. doi: 10.1080/03075079.2018.1493097

van Heerden, M., Clarence, S., and Bharuthram, S. (2017). What lies beneath: exploring the deeper purposes of feedback on student writing through considering disciplinary knowledge and knowers. *Assessment & Evaluation in Higher Education* 42 (6), 967–977. doi: 10.1080/02602938.2016.1212985

Whitworth, D. E., and Wright, K. (2015). Online assessment of learning and engagement in university laboratory practicals. *British Journal of Educational Technology* 46 (6), 1201-1213.

Yee. A. (2016). The unwritten rules of engagement: social class differences in undergraduates' academic strategies. *The Journal of Higher Education* 87 (6), 831-858. doi: 10.1353/jhe.2016.0031

Youssef, L. S. (2017). What can regular and timely student feedback tell us about the teaching and learning processes? *Reflective Practice* 18 (6), 750-771. doi: 10.1080/14623943.2017.1351350

Zimmerman, B. J. (2000). Attainment of self-regulation: A social cognitive perspective. In M. Boekaerts, P. R. Pintrich, & M. Zeidner (Eds.). *Handbook of Self-regulation* (pp. 13–39). San Diego, CA, Academic Press. doi: 10.1016/B978-012109890-2/50031-7
